# Role of Uncoupling Proteins in Cancer 

**DOI:** 10.3390/cancers2020567

**Published:** 2010-04-16

**Authors:** Adamo Valle, Jordi Oliver, Pilar Roca

**Affiliations:** Grupo Multidisciplinar de Oncología Traslacional, Institut Universitari d'Investigació en Ciències de la Salut, Universitat de les Illes Balears/Cra. Valldemossa km 7.5, E-07122, Palma de Mallorca, Illes Balears, Spain; E-Mails: adamo.valle@uib.es (A.V.); jordi.oliver@uib.es (J.O.)

**Keywords:** uncoupling proteins, cancer, proton leak, mitochondria, chemoresistance, carcinogenesis, oxidative stress

## Abstract

Uncoupling proteins (UCPs) are a family of inner mitochondrial membrane proteins whose function is to allow the re-entry of protons to the mitochondrial matrix, by dissipating the proton gradient and, subsequently, decreasing membrane potential and production of reactive oxygen species (ROS). Due to their pivotal role in the intersection between energy efficiency and oxidative stress, UCPs are being investigated for a potential role in cancer. In this review we compile the latest evidence showing a link between uncoupling and the carcinogenic process, paying special attention to their involvement in cancer initiation, progression and drug chemoresistance.

## 1. Introduction

Since Otto Warburg discovered that most cancer cells predominantly produce energy by glycolysis rather than by oxidative phosphorylation in mitochondria, much interest has been focused on the alterations of these organelles in cancer cells. Mitochondria have been shown to be key players in numerous cellular events tightly related with the biology of cancer. Although energy production relies on the glycolytic pathway in cancer cells, these organelles also participate in many other processes essential for cell survival and proliferation such as ROS production, apoptotic and necrotic cell death, modulation of oxygen concentration, calcium and iron homeostasis, and certain metabolic and biosynthetic pathways. Many of these mitochondrial-dependent processes are altered in cancer cells, leading to a phenotype characterized, among others, by higher oxidative stress, inhibition of apoptosis, enhanced cell proliferation, chemoresistance, induction of angiogenic genes and aggressive fatty acid oxidation. Uncoupling proteins, a family of inner mitochondrial membrane proteins specialized in energy-dissipation, has aroused enormous interest in cancer due to their relevant impact on such processes and their potential for the development of novel therapeutic strategies. In this review we focus on the UCPs’ currently known function and their emerging roles in the pathophysiology of cancer, paying special attention to their supposedly positive or negative role depending on the phase of cancer development. 

## 2. Mitochondria, Proton Leak and ROS

Mitochondria are microscopic organelles located in the cytoplasm of all eukaryotic cells acting as cellular powerhouses. The primary role of mitochondria is the generation of ATP through a complex process of controlled substrate degradation and oxygen consumption known as oxidative phosphorylation (OXPHOS) [1]. These organelles possess an outer and an inner membrane, the latter of which has a larger surface area, is impermeable to most molecules, and contains the large protein complexes that are necessary for energy transduction and ATP synthesis. Briefly, oxidation of reduced nutrient molecules, such as carbohydrates, lipids, and proteins, through cellular metabolism yields electrons in the form of reduced hydrogen carriers NADH+ and FADH2. These reduced cofactors donate electrons to a series of protein complexes embedded in the inner mitochondrial membrane known as the electron transport chain (ETC). These complexes use the energy released from electron transport for active pumping of protons across the inner membrane, generating an electrochemical gradient. The ultimate destiny of electrons is the reduction of molecular oxygen at complex IV yielding a molecule of water, whereas the energy, conserved as proton gradient, is used by the F0F1 ATP synthase (or complex V) to phosphorylate ADP through the return of protons into the mitochondrial matrix [2]. Thus, mitochondria are highly specialised machines that orchestrate conversions between different forms of energy, coupling aerobic respiration to phosphorylation. 

**Figure 1:**
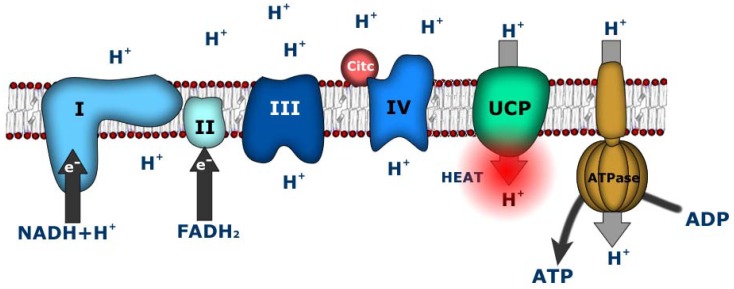
Mitochondrial oxidative phosphorylation system and uncoupling protein.

Conversion of metabolic fuel into ATP is not a fully efficient process. Some of the energy of the electrochemical gradient is not coupled to ATP production due to a phenomenon known as proton leak, which consists of the return of protons to the mitochondrial matrix through alternative pathways that bypass ATP synthase [3,4]. Although this apparently futile cycle of protons is physiologically important, accounting for 20-25% of basal metabolic rate, its function is still a subject of debate. Several different functions have been suggested for proton leak, including thermogenesis, regulation of energy metabolism, and control of body weight and attenuation of reactive oxygen species (ROS) production. Although a part of the proton leak may be attributed to biophysical properties of the inner membrane, such as protein/lipid interfaces, the bulk of the proton conductance is linked to the action of a family of mitochondrial proteins termed uncoupling proteins [5] (Figure 1). 

Mitochondria are the major sources of reactive oxygen species (ROS). Aerobic respiration involves the complete reduction of oxygen to water, which is catalysed by complex IV (or cytochrome c oxidase). Nevertheless, during the transfer of electrons along the electron transport complexes, single electrons sometimes escape and result in a single electron reduction of molecular oxygen to form a superoxide anion, which, in turn is the precursor of other ROS. Superoxide is rapidly converted to hydrogen peroxide (H_2_O_2_) spontaneously or enzymatically catalyzed by superoxide dismutase (SOD). H_2_O_2_, although it is not an oxygen free radical, can lead to the production, in the presence of ferrous iron via the Fenton reaction, of the highly reactive hydroxyl radical (·OH). 

**Figure 2:**
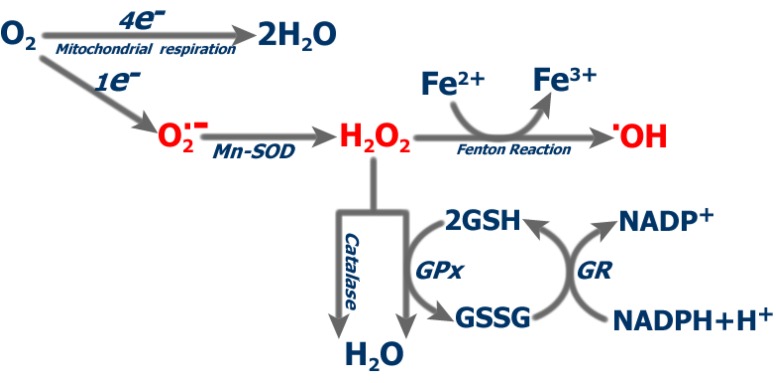
ROS production and scavenging. SOD: Superoxide dismutase; GPx: glutathione peroxidase; GR: glutahione reductase.

Cells have an elaborate antioxidant defense system to protect themselves from the attack of ROS. This system includes enzymes such as superoxide dismutase, catalase and several peroxidases, as well as antioxidant compounds (vitamins C, E, glutathione), which independently or in cooperation contribute to neutralize ROS (Figure 2). When cellular production of ROS overwhelms the overall antioxidant defences, free radicals may escape and exert their deleterious effects. This situation, called oxidative stress, is supposed to be responsible for the accrual of cellular damage during lifetime, thereby playing a role in the etiogenesis and course of numerous pathologies and in aging [6,7]. Macromolecules within the mitochondria are more prone to ROS-induced damage due to their physical proximity to the source of ROS. In addition, mitochondrial DNA which lacks the protective shields of histones and also has limited DNA-repairing systems is especially vulnerable to such damage. It is worth noting that the damage exerted by ROS on mitochondrial DNA may lead to a higher degree of mitochondrial dysfunction and, in turn, to higher ROS production, leading to a vicious cycle of ROS amplification.

Nevertheless, ROS should not be seen only as negative or damaging molecules. It is worth noting that the rapidly-produced, short-lived, and highly diffusible ROS fit the characteristics of a second messenger molecule perfectly. In fact, although ROS cause damage, low levels of ROS are considered to participate in cell signaling processes such as cell proliferation, inflammation, apoptosis and phagocytosis [8].

## 3. Uncoupling Proteins

**Figure 3:**
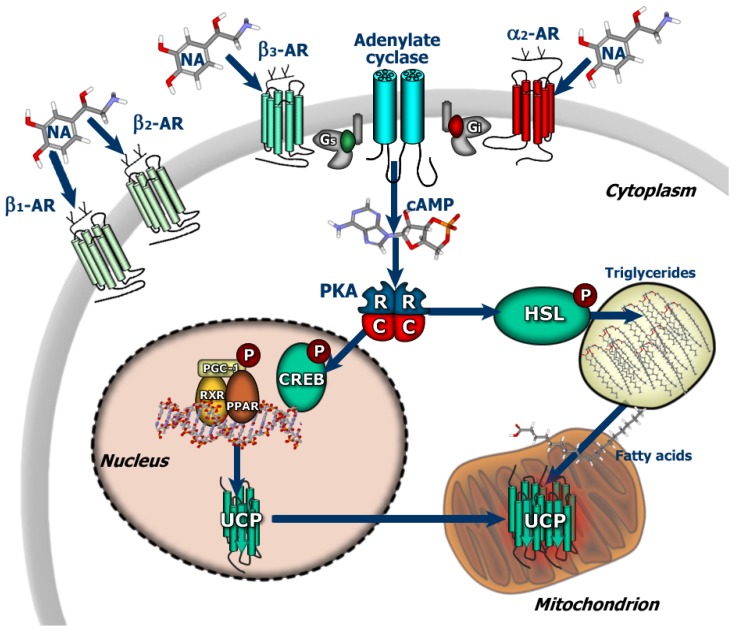
Adrenergic control of thermogenesis in brown adipose tissue. AR: adrenergic receptor; HSL: hormone-sensitive lipase.

The first member of the uncoupling protein family, currently termed UCP1, was discovered in brown adipose tissue (BAT) [9]. BAT is a particular form of adipose tissue whose main function is nonshivering thermogenesis. It has been classically demonstrated to be present in hibernators, small mammals and infants at birth, nevertheless recent evidence have suggested its presence and activity also in adult humans [10]. In contrast to white adipocytes, brown adipocytes possess numerous triglyceride droplets, are directly innerved by the sympathetic system, and have a great amount of mitochondria characterized by a highly developed inner membrane. Activation of brown adipocytes by cold-induced release of noradrenaline is immediately followed by increased respiration and heat production. The tissue is located near large blood vessels, which, on the one hand, assures oxygen and nutrient supply for respiration and, on the other hand, an efficient system to warm blood and carry heat to the heart and brain [11,12,13]. Mitochondria from brown adipocytes have large amounts of UCP1 which promotes proton leakage independent of ADP phosphorylation, thus uncoupling respiration from ATP synthesis, thereby dissipating oxidation energy in the form of heat [14]. Activation of thermogenesis is commanded by the central nervous system and the sympathetic fibers innervating each brown adipocyte. The noradrenaline released by these fibers binds to several types of adrenergic receptors on the surface of the brown adipocytes and activates production of cyclic AMP inside the cell, which in turn activates lipolysis and oxidation of fatty acids by mitochondria. In the short-term, UCP1 activity is directly activated by the fatty acids released by lipolysis, whereas, in the long-term, cAMP induces the expression of UCP1, such as occurs during cold-acclimation (Figure 3). On the other hand, purine nucleotides such as ADP and GDP are able to bind and inhibit UCP1, which could be a negative regulatory feed-back mechanism aimed to avoid uncoupling in energy demanding conditions. The mechanism of action of UCP1 is subject to debate: some scientists believe it is a proton transporter, whilst others assert that it returns anionic fatty acids to the intermembrane space, after they have crossed the membrane in protonated form, which also results in a net translocation of protons and therefore in uncoupling.

In 1995, a second known uncoupling protein (termed PUMP) was found in plants and since 1997 four new UCP homologs (UCP2 to UCP5) have been identified in humans and rodents. In contrast to the BAT specific UCP1, UCP homologs have a wider tissular expression. UCP2 is the most ubiquitous, as it is present in many tissues, such as adipose tissue, muscle, heart, kidney, digestive tract, brain, spleen and thymus whereas UCP3 expression is principally restricted to human and rodent skeletal muscle and rodent heart and BAT [15,16,17]. UCP4 and 5 are the most recently discovered UCP homologs and their expression is mainly restricted to brain, although some UCP5 has been found in other tissues such as testis and pituitary [18,19,20]. Thus, considering that most of the tissues in organisms express at least one or even two homologs of UCP, these proteins are likely to play a physiological role other than adaptive thermogenesis. Moreover, the identification of UCPs in plants, fungi and protozoa indicates that the UCPs form an ancient and conserved family. Regarding phylogenesis, several studies have suggested that UCP4 is the closest homolog to the ancestral prototype of UCP [21]. UCP2 appears to have evolved late in phylogenesis whereas UCP3 and, curiously, the firstly discovered UCP1, would be the most recent evolutive forms of the protein [21]). The fact that the earliest UCP isoform would be UCP4, which is the only one expressed in *C. elegans* and co-expressed with UCP5 in *Drosophila*, is quite intriguing considering that in humans, rats, and mice, its expression is restricted to brain. In any event, what UCPs’ phylogenesis makes clear is that any role that emerged with these proteins is important enough to persist.

The reactions catalyzed by UCP homologs and their physiological roles are still under debate, with the literature containing contrasting results (reviewed in [22]). UCP2 and UCP3 have 59 and 57 % identity, respectively, with UCP1, and 73% identity with each other [23]. Due to their homology to UCP1 and their distribution in several mammalian tissues, it was initially postulated that UCP2 and UCP3 are also thermogenic and involved in regulation of energy expenditure and body weight [24]. Enforced overexpression of UCP2 and UCP3 in yeast has demonstrated that, similar to UCP1, each of these homologs can reduce the mitochondrial membrane potential and promote thermogenesis [15]. However, such uncoupling was generally obtained when UCP2 or UCP3 was expressed at a much higher level than that measured in tissues [25,26,27]. On the other hand, Ucp2 or Ucp3 knockout mice maintain their body temperature in a cold environment. Therefore, although there are data in favor of an uncoupling activity of these UCP homologs, unlike UCP1, UCP2 and UCP3 they are not involved in adaptive thermogenesis. Nevertheless, several genetic studies have pointed to an association between some Ucp2 polymorphisms and basal energy expenditure [28], which suggests an involvement in controlling resting metabolic rate [29]. In addition, Ucp2 gene is in proximity to a cluster of genes related to energy homeostasis and obesity [15,30] and its promoter region contains several response elements that may explain fatty acid responsiveness and regulation of Ucp2 in response to obesity, fasting and other conditions [31].

Similarly, UCP3 has also been linked to fatty acid metabolism. Ucp3 expression is elevated during states that are associated with increased fat metabolism such as fasting [32,33], acute exercise [34,35] and high-lipid diet [36,37]. Consistently, skeletal muscle mitochondria of mice overexpressing Ucp3 show increased fatty acid oxidation rates and decreased intramuscular fat stores [38,39]. It has been suggested that the function of UCP2 and UCP3 is to export fatty acid anions outside of the mitochondrial matrix when there is a large excess of fatty acids inside mitochondria [40]. This transport of fatty acids out of the mitochondria would, on the one hand, protect mitochondria from the toxic effect of excessive amounts of fatty acid anions and, on the other hand, allow the reactivation of these fatty acids by cytosolic acyl-CoA synthetases, allowing continued rapid fatty acid oxidation and thereby preventing mitochondrial damage [41,42]. Although the fatty acid anion export hypothesis has several lines of supportive evidence, data from Ucp3 knockout mice are controversial, showing no effect or even reduced fatty acid oxidation [23,43]. 

Another function related to energy homeostasis was found for UCP2 in the regulation of insulin secretion in beta pancreatic islets [44]. The beta-cells sense glucose through its catabolism, increase the ATP/ADP ratio which closes the ATP-sensitive potassium channel, causing plasma membrane depolarization, which opens voltage-sensitive calcium channels. Subsequently, the increase in Ca^2+^ anions into the cytosol triggers insulin secretion [44,45]. UCP2, by means of its proton-leak activity, decreases ATP production and, thus, impairs glucose-stimulated insulin secretion [46]. In support, Ucp2 knockout mice have mitochondria with higher ATP levels and increased insulin secretion in response to glucose.

One of the most interesting functions attributed to UCPs is their ability to decrease the formation of mitochondrial ROS. Mitochondria are the main source of ROS in cells. Superoxide formation is strongly activated under resting (state 4) conditions when the membrane potential is high and the rate of electron transport is limited by lack of ADP and Pi [47]. Thus, there is a well established strong positive correlation between membrane potential and ROS production. At high membrane potential, a small increase in membrane potential gives rise to a large stimulation of ROS production [48], whereas a small decrease in membrane potential (10 mV) is able to inhibit ROS production by 70% [49,50]. Therefore, mild uncoupling, *i.e.*, a small decrease in membrane potential, has been suggested to have a natural antioxidant effect [51]. Consistent with such a proposal, the inhibition of UCPs by GDP in mitochondria has been shown to increase membrane potential and mitochondrial ROS production [52,53]. The loss of UCP2 or UCP3 in knockouts yielded increased ROS production concurrent with elevated membrane potential specifically in those tissues normally expressing the missing protein [54,55]. These findings suggest that these proteins maintain a normal membrane potential below the threshold of excessive ROS generation, yet with an undetectable effect on body weight or whole-animal metabolic rate [54,55,56,57]. The hypothesis of UCPs as an antioxidant defense has been strongly supported by the fact that these proteins have been shown to be activated by ROS or by-products of lipid peroxidation, showing that UCPs would form part of a negative feed-back mechanism aimed to mitigate excessive ROS production and oxidative damage [58,59,60,61]. Several experiments support the idea that superoxide activates UCPs through a free radical chain reaction which forms reactive aldehydes such as hydroxynonenal (HNE) [47,62]. Thus, HNE added to isolated mitochondria decreases ROS production and membrane potential whereas the inhibition of UCPs by GDP protects ROS production against the effect of HNE [62]. Alternatively, other authors have proposed that the antioxidant activity of UCPs would consist in translocate fatty acid peroxides, instead of protons, from the inner to the outer membrane leaflet, contributing to protect mitochondrial DNA from oxidative damage [63,64].

UCP4 and UCP5 are the least known homologs. Their brain abundance is consistent with an important physiological relevant function in the CNS. Ectopic expression of these proteins in different cell lines results in higher state 4 oxygen consumption and reduced mitochondrial membrane potential and ROS levels [20,65,66,67,68,69]. Similarly to UCP2 and UCP3, roles in antioxidant protection, metabolic reprograming and thermogenesis have been proposed for these homologs in the brain [70,71].

## 4. ROS and Cancer

**Figure 4:**
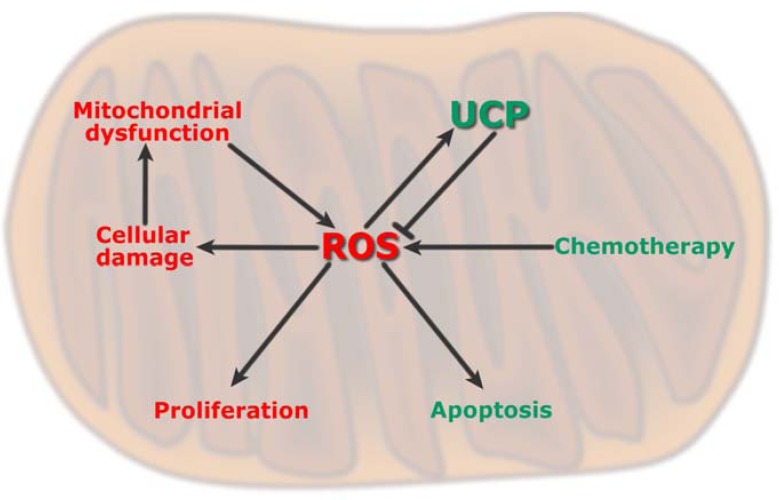
Role of ROS in control of proliferation and apoptosis.

ROS are thought to play multiple roles in tumor initiation, progression and maintenance, eliciting cellular responses that range from proliferation to cell death [72,73,74] (Figure 4). In normal cells, ROS play crucial roles in several biological mechanisms including phagocytosis, proliferation, apoptosis, detoxification and other biochemical reactions. Low levels of ROS regulate cellular signaling and play an important role in normal cell proliferation [75,76]. 

During initiation of cancer, ROS may cause DNA damage and mutagenesis, while ROS acting as second messengers stimulate proliferation and inhibit apoptosis, conferring growth advantage to established cancer cells [72,73,74]. Cancer cells have been to have increased ROS levels [75,77]. One of the functional roles of these elevated ROS levels during tumor progression is constant activation of transcription factors such as NF-kappaB and AP-1 which induce genes that promote proliferation and inhibit apoptosis [78,79]. In addition, oxidative stress can induce DNA damage which leads to genomic instability and the acquisition of new mutations, which may contribute to cancer progression as well [80].

**Figure 5:**
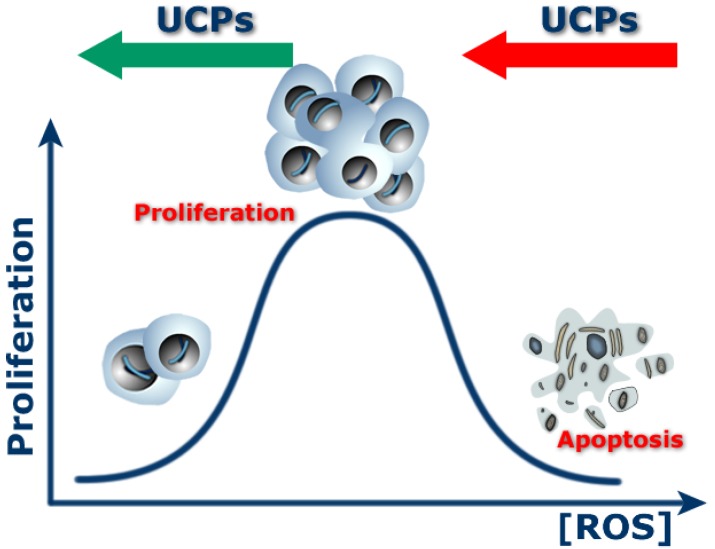
Effect of uncoupling proteins on proliferation and apoptosis in relation to ROS levels.

ROS are also essential mediators of apoptosis which eliminates cancer and other cells that threaten our health [81,82,83,84,85,86]. Many chemotherapeutic drugs and radiotherapy are aimed at increasing ROS levels to promote apoptosis by stimulating pro-apoptotic singaling molecules such as ASK1, JNK and p38 [87,88]. Because of the pivotal role of ROS in triggering apoptosis, antioxidants can inhibit this protective mechanism by depleting ROS [89,90]. Thus, antioxidant mechanisms are thought to interfere with the therapeutic activity of anticancer drugs that kill advanced stage cancer cells by apoptosis. Since UCPs are powerful modulators of mitochondrial ROS production, these proteins play pivotal but different roles depending on the stage of cancer (Figure 5). Thus, in normal cells, mild uncoupling could be a protective mechanism for buffering excessive ROS production and mutagenesis, whereas in advanced cancer cells, overexpression of UCPs may be a selected mechanism to induce chemoresistance. Below, we focus on the potential role of UCPs in such stages of cancer. 

## 5. Roles of UCPs in Cancer Initiation

Oxidative stress has been postulated to play a role in cancer initiation. In fact, several ROS and lipid peroxidation byproducts, such as malondialdehyde (MDA), 4-hydroxynonenal (4-HNE), quinones and alkenals are all known to produce DNA damage, directly contributing to the carcinogenic process. UCPs, by decreasing membrane potential, are negative regulators of ROS production. Taking into account the genotoxic effect of ROS, it is tempting to speculate that one of the predictable consequences of UCPs function may be to prevent cancer. Nevertheless, there is a lack of literature in which carcinogenesis has been analyzed in relation to UCPs.

Primary evidence of a link between UCPs and carcinogenesis can be deduced from the low incidence of BAT-derived tumors, also called hibernomas. In contrast to a lipoma, which originates from white adipose tissue and is among the most common soft-tissue tumors, hibernoma is listed among the rarest [91,92]. Although BAT is a metabolically active tissue, with oxygen consumption rates similar to brain, liver or muscle, its tumor incidence is extremely low, suggesting that the prominent feature of uncoupled respiration may be less genotoxic. This assumption is in agreement with the ROS lowering activity of UCPs. Nevertheless, other mechanisms such as better DNA repairing systems or antioxidant defenses, although lacking supportive evidence so far, cannot be discarded.

Interesting data could be obtained from studies analyzing carcinogenesis in animal models with altered UCP expression. Studies on mice overexpressing or deficient in UCP2- and/or UCP3, nevertheless, have been mainly addressed to study the involvement of UCPs in aging and lifespan [93,94]. A role for UCPs regulating lifespan has been strongly suggested since mitochondrial dysfunction and ROS production are at the heart of the aging process. Although data are limited, some of these studies hint at a link between UCPs and tumor incidence. It has to be taken into account that cancer is an age associated disease and one of the main causes of death in housed aged rodents [95]. Speakman and colleagues studied the association between metabolic intensity and longevity across individuals from a single strain of mice. They found that mice in the upper quartile of metabolic rate, showing greater resting oxygen consumption and UCP3 in skeletal muscle, lived 36% longer than mice in the lowest quartile [96]. Unfortunately, these authors did not perform an autopsy to determine the cause of death, but pointed to the incidence of several cases of abdominal tumours [96]. This and other studies [97,98,99] support the uncoupling-to-survive hypothesis proposed by Brand [100]. This hypothesis states that increased uncoupling leads to greater oxygen consumption, lower ROS production and as a result, lower oxidative damage and increased lifespan. In agreement with this hypothesis recent studies by Andrew and Horvath showed that UCP2−/− mice have a significantly shorter survival age [93]. Mice overexpressing human UCP2 (hUCP2-Tg) have a delayed time of first death, even though they have the same survival age as their wild-type control. Moreover, to assess the impact of UCP2 on ROS production and subsequent survival age, these authors crossed the UCP2 deficient mice with mice knocked out for superoxide dismutase 2 (SOD2), producing sod2−/− Ucp2−/− genotypes. Sod2−/− mice die at 3 weeks of age from increased mitochondrial oxidative injury mainly in the central nervous system and heart [101]. The double knockout mice (sod2−/−, ucp2−/−) had a significantly reduced lifespan when compared to sod2−/−; ucp2 wild-type mice, whereas the crossing of sod2−/− with hUCP2-Tg mice increased the survival age compared to wild-type controls. These results suggest that the ability of UCP2 to increase lifespan is mediated by decreased ROS production and oxidative stress. Nevertheless, neither did this study analyze the cause of death of the mice. In contrast, McDonald *et al*. failed to find increased mean survival in UCP2−/− and UCP3−/− mice, whereas overexpression of both UCP2 and UCP3 induced a slight increase in lifespan [94]. Fortunately, these authors performed histopathological analyses of several tissues of the dead mice. Although the authors state that their pathological analyses were presented only to provide a general understanding of the types of lesions during aging and were not intended to determine the cause of death, their valuable data showed a higher incidence of tumours in the UCP2−/− UCP3−/− (1.53 neoplasic lesions/mouse) compared to wild-type control (1.01 lesions/mouse), and a lower incidence in transgenic mice overexpressing UCP2 and UCP3 (0.64 lesions/mouse). Although these data are not sufficient to support the role of UCPs in tumourigenesis, the ability of mild uncoupling to avoid ROS formation, gives a reasonable argument to hypothesize about a role for UCPs in cancer prevention [102]. Further research aimed at a possible protective function of UCPs, using mice with modified UCP expression living under normal conditions, is required to statistically confirm the influence of UCPs on carcinogenesis through modulation of ROS production.

One of the handicaps of such studies is that deletion of UCPs in non-stressed housed animals may cause such subtle changes in oxidative stress to require long-term periods in order to accumulate enough damage to induce cancer. In fact, the phenotype of Ucp2−/− mice remains relatively unremarkable, unless these animals are challenged by metabolic stress, infection or surgery [54,103,104]. Therefore, it is worth considering that UCPs may have a greater impact on cancer incidence under such stress inducing conditions. Consistently, Derdák *et al*. showed that Ucp2−/− mice treated with the carcinogen azoxymethane were found to develop more aberrant crypt foci and colon tumours than Ucp2+/+ in relation with increased oxidative stress and enhanced NF-kappaB activation [105]. Thus, a total of four tumours were found in ten two-year-old UCP2-ablated mice, but not a single wild-type littermate had developed a tumour by that age [105]. 

From a physiological point of view, it is worth noting that hormonal modulation of UCPs could also be a factor involved in hormone-induced carcinogenesis. In this sense, studies in our lab have suggested that UCPs may also be underlying the carcinogenic effect of estrogens in breast [106]. Estrogens (E2) are a major risk factor for breast cancer initiation and progression [107,108,109]. Although E2 have been shown to act as an antioxidant in several tissues [110,111,112], there is strong controversy regarding their oxidative role in E2-dependent tissues such as breast, ovary or uterus [113,114,115,116,117,118,119,120]. Recently we have shown that E2 are able to decrease several UCP homologs in the ER positive MCF7 breast cancer cell line [106]. Mitochondria isolated from E2 treated cells showed a higher membrane potential and ROS production whereas the ability of GDP to induce ROS was decreased. Although further confirmation in non-transformed cells and *in vivo* mammary gland is needed, the hypothesis of breast-specific estrogenic repression of uncoupling proteins is quite tentative since a loss of uncoupling contributes to E2-induced cell proliferation both by an increase in mitochondrial efficiency at ATP synthesis as well as by increasing ROS acting as mitogenic signals. This hypothesis may contribute in part to explain why elevated lifetime estrogen exposure is a well-known major risk factor for breast cancer. On the whole, these pioneer studies offer new clues of the involvement of UCPs in cancer initiation. Nevertheless, growing evidence of a protective effect of UCPs against ROS and the strong association between oxidative stress, mutagenesis and carcinogenesis, require further studies addressed to investigate the ways that UCPs may impact carcinogenesis. 

## 6. Roles of UCPs in Cancer Progression

The growth of a tumor from a single genetically altered cell is a stepwise progression requiring the alterations of several genes which contribute to the acquisition of a malignant phenotype. Such genetic alterations are positively selected when in the tumor, they confer a proliferative, survival or treatment-resistance advantage for the host cell. In addition, several mutations, such as those silencing tumour-suppressor genes, trigger the probability of accumulating new mutations, so the process of malignant transformation is progressively self-accelerated. Considering the ability of UCPs to modulate mutagenic ROS, as well as mitochondrial bioenergetics and membrane potential, both involved in regulation of cell survival, an interesting question is whether UCPs can be involved in the progression of cancer. Interestingly, UCP2 expression has been shown to be increased in several hepatocellular cancer and cell lines, oxyphilic thyroid tumors and human colon cancer [121,122,123]. UCP2 has been observed to correlate with the degree of neoplastic changes in colon [122]. Consistently, another study showed a relationship between the degree of mitochondrial dysfunction in several transformed colonic cells and the levels of UCP2 and UCP5 [124]. ROS levels in such studies showed a positive correlation with UCP2 expression [122,124], suggesting that elevated UCP levels may be a response to increased oxidative stress. 

Nevertheless, as discussed below, increased expression of UCPs in cancer cells may confer several advantages. Among these, increased uncoupled respiration may be a mechanism to lower cellular oxygen concentration and, thus, alter molecular pathways of oxygen sensing such as those regulated by hypoxia-inducible factor (HIF). In normoxia, the alpha subunit of HIF-1 is a target for prolyl hydroxylase, which makes HIF-1alpha a target for degradation by the proteasome. During hypoxia, prolyl hydroxylase is inhibited since it requires oxygen as a cosubstrate [125]. Thus, hypoxia allows HIF to accumulate and translocate into the nucleus for induction of target genes regulating glycolysis, angiogenesis and hematopoiesis [126,127]. By this mechanism, UCPs activity may contribute to increase the expression of genes related to the formation of blood vessels, and thus promote tumor growth. 

Nevertheless, it is paradoxical that these cancer cells have increased ROS levels despite their greater levels of UCPs. Targeted manipulation of mitochondrial ROS levels in cancer cells could be a good strategy to study the cause-and-effect link between increased oxidative stress and UCPs overexpression.

## 7. Roles of UCPs in Cancer Energy Metabolism

Over half a century ago, Otto Warburg discovered that one of the bioenergetic signatures of cancer cells is the acquisition of a glycolytic phenotype even in the presence of oxygen [128]. At first sight, this observation of an increased rate of aerobic glycolysis, known as the Warburg effect, appeared counterintuitive considering the lower efficiency of glycolytic ATP production in contrast with the high rates of energy demand in rapidly growing cancer cells. However, the Warburg effect has been reproduced numerous times and confirmed by independent proofs, suggesting that cancer cells really benefit from this metabolic shift. Although at first Warburg attributed the increased dependence of cancer cells on glycolysis to alterations to the oxidative capacity of mitochondria, it has been shown by others that the machinery of oxidative phosphorylation often remains intact and functional in such cells, which leads to question the hypothesis of mitochondrial injuries as a cause of the Warburg effect [129]. Lynen and colleagues proposed that the root of the Warburg effect is not in the inability of mitochondria to carry out respiration, but rather would rely on their incapacity to synthesize ATP in response to membrane potential [128]. 

The ability of UCPs to uncouple ATP synthesis from respiration and the fact that UCP2 is overexpressed in several chemoresistant cancer cell lines and primary human colon cancers have lead to speculate about the existence of a link between UCPs and the Warburg effect [130]. As mentioned above, uncoupling induced by overexpression of UCP2 has been shown to prevent ROS formation, and, in turn, increase apoptotic threshold in cancer cells, providing a pro-survival advantage and a resistance mechanism to cope with ROS-inducing chemotherapeutic agents. Glycolytic ATP production may concile the advantages of UCP2 overexpression with the need of energy to sustain rapid cell growth [130]. 

Besides increased rates of ATP production, cancer cells require enhanced biosynthesis to sustain cell growth. Mitochondrial Krebs cycle is one of the sources for these anabolic precursors. Nevertheless, the export of these metabolites to cytoplasm for anabolic purposes involves the replenishment of the cycle intermediates by anaplerotic substrates such as pyruvate and glutamate. Thus, glycolysis-derived pyruvate, as well as alpha-ketoglutarate derived from glutaminolysis, may be necessary to sustain anaplerotic reactions [131]. At the same time, to keep Krebs cycle functional, the reduced cofactors NADH and FADH2 would have to be re-oxidized, a function which relies on the mitochondrial respiratory chain. Once again, uncoupling may be crucial for cancer cell mitochondrial metabolism, allowing Krebs cycle to be kept functional to meet the vigorous biosynthetic demand of cancer cells. 

Last but not least, it is important to take into account that UCPs are largely associated with fatty acid oxidation [132]. Several cancer cells resistant to chemotherapeutics and radiation often exhibit higher rates of fatty acid oxidation [40] and it has been observed that inhibition of fatty acid oxidation potentiates apoptotic death induced by chemotherapeutic agents [133]. These findings are in agreement with the proposed need of fatty acid for the activity of UCPs, suggesting that the lack of these potential substrates or activators would decrease uncoupling activity, subsequently increasing membrane potential, ROS production and therefore lowering apoptotic threshold. 

Recently, Bouilllaud *et al*. have proposed that the presence of UCP2/3 may act decreasing the affinity of mitochondria for pyruvate, instead of uncoupling, probably by means of a uniport for anionic pyruvate [134]. This hypothesis may explain some reported modifications in glucose sensing linked to UCP2 activity that are not accompanied by clear evidence of uncoupling. Nevertheless, further evidence of the ability of UCP2/3 to transport pyruvate is necessary to give robustness to this hypothesis. 

## 8. Roles of UCPs in Cancer Cachexia

Cachexia is a wasting syndrome characterized by weakness, weight and fat loss, and muscle atrophy which is often seen in patients with advanced cancer or AIDS. Cachexia has been suggested to be responsible for at least 20 % of cancer deaths [135] and also plays an important part in the compromised immunity leading to death from infection. The imbalance between energy intake and energy expenditure underlying cachexia cannot be reversed nutritionally, which indicates the existence of a fundamental pathology. It has been proposed that alterations leading to high energy expenditure, such as excessive proton leak or mitochondrial uncoupling, are likely mechanisms underlying cachexia. In fact, increased expression of UCP1 in BAT and UCP2 and UCP3 in skeletal muscle have been shown in several murine models of cancer cachexia [136,137]. The induction of UCP1 was firstly considered an adaptive response to cancer induced hypothermia. Similarly, the increased expression of UCP2 and UCP3 in muscle of cachexic animals, was attributed to increased levels of fatty acids as a result of lipolysis induced by reduced food intake [136]. Nevertheless, implantation of a fast growing tumour in mice (Lewis lung carcinoma) resulted in a clear cachectic state accompanied by a significant increase in both UCP2 and UCP3 gene expression in skeletal muscle and heart, whithout a rise in circulating fatty acids or a decrease in food intake, questioning hyperlipaemia as the only factor controlling UCP2-3 expression in cancer cachexia [138]. The discovery of the lipid mobilizing factor (LMF), a small protein also know as zinc-α2-glycoprotein (ZAG) which is produced by cachexia-inducing tumours, established a paracrine link between cachexic tumours and UCP expression. ZAG has been shown to increase UCP1 expression in primary cultures of BAT, as well as expression of UCP2 and UCP3 in murine myotubes [139]. Such induction of UCP1 and UCP2 has been shown to be mediated through β3-adrenergic receptor, whereas induction of UCP3 appears to require mitogen-activated protein kinase. Besides the production of ZAG in the tumor, it is also produced by white adipose tissue and BAT, with this expression increased during cachexia. These findings suggest an autocrine role of adipocyte-derived-ZAG in the induction of both lipolysis and UCP expression [140]. Although UCPs are not the only player in the complex process of cachexia, their potential contribution and therapeutic prospects deserve further research.

## 9. Roles of UCPs in Chemoresistance

Cancer cells acquire drug resistance as a result of selection pressure dictated by unfavorable microenvironments. Although mild uncoupling may clearly be useful under normal conditions or under severe or chronic metabolic stress such as hypoxia or anoxia, it may be a mechanism to elude oxidative stress-induced apoptosis in adavanced cancer cells. Several anti-cancer treatments are based on promotion of ROS formation, to induce cell growth arrest and apoptosis. Thus, increased UCP levels in cancer cells, rather than a marker of oxidative stress, may be a mechanisms confering anti-apotptotic advantages to the malingant cell, increasing their ability to survive in adverse microenvironments, radiotherapy and chemotherapy. Supportive evidence comes from ectopic expression experiments, in which HepG2 human hepatoma cells overexpressing UCP2, reduced oxidative stress and increased resistance to apoptosis induced by menadione or hypoxia/ reoxygenation [141]. Similarly, ectopic UCP2 expression in HCT116 human colon cancer cells decreased apoptosis induced by UV radiation and chemotherapy by mechanisms involving modulation of the p53 pathway, a pivotal tumor suppressor [142]. Besides UCP2, neural cells express the brain uncoupling protein UCP4 have also been reported to exhibit increased cellular resistance to toxicity induced by 3-nitropropionic acid, a mitochondrial complex II inhibitor that compromises cellular bioenergetics [143]. Consistent with these findings, other several drug-resistant sublines of cancer cells derived from leukemia and melanoma have been shown to overexpress UCP2 [40]. Thus, UCPs appear to play a permissive role in tumor cell survival and growth. 

On the whole, these works suggest that expression of UCPs promote bioenergetics adaptation and cell survival. UCPs appear to be critical to determine the sensitivity of cancer cells to several chemotherapeutic agents and radiotherapy, interfering with the activation of mitochondria driven apoptosis.

## 10. Therapeutic Prospects of UCPs

UCPs are attracting growing interest as potential therapeutic targets in a number of important diseases such as obesity, diabetes, cardiovascular and neurodegenerative disorders and cancer [144]. With regard to obesity, the discovery of UCP1 homologs in the 90s was believed to be promising to find a treatment for obesity. Nevertheless, phenotypes of mice with inactivated Ucp2 or Ucp 3 genes are not related to defective body weight regulation, indicating that a strategy based only on targeting UCP2 or UCP3 would not be able to counteract obesity. More promising is the effect of reduced expression of UCP2 on improved insulin secretion in the pancreas [145]. However, the indication that UCP2 protects against tumours implies that a general reduction of UCP2 activity might have undesirable side effects [102]. In contrast, in models of neurodegenerative and cardiovascular disease, experimental evidence suggests that an increased expression and activity of UCP2 has a beneficial effect on disease progression, implicating a potential therapeutic role for UCP2 [144]. 

Cancer-specific mitochondrial alterations and bioenergetics may be taken advantage of for the development of two different types of antineoplastic agents. A first approach would target glycolysis and/or revert the Warburg phenomenon, returning the energy producing responsibility of the cell to mitochondria, whereas the second approach would aim at inducing apoptosis by targeting mitochondrial proteins and membranes [146]. Regarding the former, the generic drug dichloroacetate has generated much interest since, by inhibiting pyruvate dehydrogenase kinase, it increases the flux of pyruvate into the mitochondria, promoting glucose oxidation over glycolysis. This reversion of the Warburg effect has been shown to increase apoptosis and results in suppression of tumour growth both *in vitro* and *in vivo* [147,148,149]. This mechanism involving inhibition of glycolysis by metabolic modulators could be especially effective in tumours overexpressing UCP2, since their uncoupled mitochondria are unable to produce compensatory ATP from respiration [148]. Thus, the observed overexpression of UCP2 in cancer cells, but not in normal cells, may provide a plausible molecular mechanism by which acetoacetate spares normal cells but suppresses growth in cancer cells.

Regarding the second strategy, UCP2 could be a potential target of drugs designed to fight against therapy-resistant cancers. Inhibition of UCP2, by enhancing ROS production, may increase susceptibility of cancer cells to apoptosis. In fact, UCP2 silencing results in higher rates of activation of apoptotic pathways in leukemia cells transfected with UCP2 siRNA [150]. Recently, UCP2 downregulation and the resultant increased oxidative stress have been shown to underly the mechanisms of taxol-induced apoptosis of melanoma cells [151]. Given the role of UCPs in the metabolic shift associated with increased fatty acid oxidation, it is also interesting that pharmacologic inhibition of fatty acid oxidation has been shown to potentiate apoptosis induced by a variety of chemotherapeutics in cancer cell lines [133,152], as well as palmitate-induced apoptosis in hematopoietic cells [153]. Although the involvement of UCPs in this induction of apoptosis was not studied, it is tempting to speculate that targeting fatty acid oxidation may be a strategy to inhibit UCPs activity. 

The development of molecular inhibitors or antagonists of UCP2 activity has become an interesting field of research. Purine nucleotides (ATP, ADP, GTP and GDP) are the natural inhibitors of UCPs, however, these molecules are not cell permeable and are therefore unable to inhibit UCPs when added to intact cells or animals. The discovery of a UCP2 inhibitor capable of working in such models would be an extremely useful tool for the above mentioned pharmacological purposes. Recently, the naturally occurring agent genipin has been found to inhibit UCP2 in several cell types, including pancreatic islets, 3T3L1 adipocytes and cardiomiocytes [154,155,156]. Genipin is the active compound found in the gardenia fruit extract which has been used in Traditional Chinese Medicine to relieve the symptoms of type 2 diabetes. In fact, in beta pancreatic cells, genipin increases mitochondrial membrane potential, increases ATP levels, closes plasma membrane K_ATP_ channels, and stimulates insulin secretion in a UCP2-dependent manner [155]. Importantly, genipin has been reported to induce apoptotic cell death in human hepatoma cells and prostate cancer cells via increased ROS production and JNK activation of mitochondrial pathway [157,158]. Similar results in HCT116 human colon cancer cells were observed by Mark and Baffy [159] . Although genipin is an excellent natural cross-linker for proteins, the cross-linking activity of genipin appears not to be required for its biological activity as UCP inhibitor since a genipin derivative lacking the crosslinking activity maintains its ability to inhibit UCP2-mediated proton leak [155]. On the whole, these preliminary findings suggest that genipin or a derivative could be a useful treatment to inhibit UCPs in several diseases in which this protein could play a critical role. Nevertheless, inhibition of UCPs, has to be considered as a strategy aimed at promoting the effects of other therapeutic agents, contributing to avoid the anti-apoptotic shield that UCP2 confers to cancer cells.

## 11. Conclusions

As reviewed herein, UCPs are key players in the intersection of cellular energy metabolism, ROS production and fatty acid oxidation, crucial events that contribute to determine cell survival and proliferation. In the last few years, growing evidence has been found supporting the importance of UCPs in the metabolic reprogramming and development of chemo-resistance of cancer cells. From a therapeutic viewpoint, inhibition of glycolysis in UCP2 expressing tumours or specific inhibition of UCP2 are, respectively, attractive strategies to target the specific metabolic signature of cancer cells or enhance the effectiveness of ROS-inducing agents. Further research will provide significant evidence of the feasibility of these kinds of strategies for the treatment of cancer.
